# The First Example of Hetero‐Triple‐Walled Metal–Organic Frameworks with High Chemical Stability Constructed via Flexible Integration of Mixed Molecular Building Blocks

**DOI:** 10.1002/advs.201500283

**Published:** 2015-12-03

**Authors:** Dan Tian, Jian Xu, Zhao‐Jun Xie, Zhao‐Quan Yao, Deng‐Lin Fu, Zhen Zhou, Xian‐He Bu

**Affiliations:** ^1^ School of Materials Science and EngineeringSchool of ChemistryTKL of Metal‐ and Molecule‐Based Material ChemistryInstitute of New Energy Material ChemistryCollaborative Innovation Center of Chemical Science and Engineering (Tianjin)Nankai UniversityTianjin300071P.R. China

**Keywords:** cobalt, metal–organic frameworks, mixed molecular building block, multiwalled frameworks, synthetic methodology

## Abstract

**An unprecedented 3D hetero‐triple‐walled metal‐organic framework is** obtained by straightforward elaboration of the mixed molecular building block (MBB) strategy. In this approach, multiple individual flexible and rigid MBBs are integrated into one composite building block as separate layers, which are of the same shape but different sizes. This MOF shows exceptional water stability and the application of Li‐ion battery electrodes.

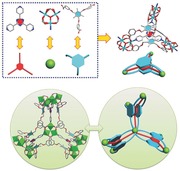

Metal‐organic frameworks (MOFs) are highly impressive for their remarkable diversity in terms of compositions and structures,^1^, ^2^, ^3^, ^4^, ^5^ which has been regarded as a driving source of their great potential in various applications, including gas storage, separation, catalysis, sensing, and recognition, and drug delivery, among others.^6^, ^7^, ^8^, ^9^, ^10^ Thus, novel and unique crystalline MOF structures have been vigorously pursued in the past decade, leading to widespread efforts by increasing the level of structural complexity to attain more fascinating architectures, such as cage‐in‐cage,^11^, ^12^ polyhedron,^13^, ^14^ high nuclearity,^15^, ^16^ and etc.^17^, ^18^ Among them, a rather straightforward way of systematically increasing the structural complexity, that is, to fabricate MOFs in a multiwalled organization has however been less explored. While an analogous concept has been extensively studied in the field of carbon nanotubes,^19^, ^20^ most existing 3D MOFs are invariably constructed with molecular building blocks (MBBs) connected by single metal centers or clusters (e.g., **Scheme** **1**a), loosely defined here as the single‐walled MOFs.^21^, ^22^, ^23^, ^24^ Essentially, the multiwalled MOFs not only possess more aesthetic appeal, but also exhibit a high degree of structural robustness owing to the increased wall thickness,^25^, ^26^, ^27^, ^28^, ^29^ the latter significantly improving the applicability of MOFs in many fields. Thus, a reliable and facile strategy to construct the promising multiwalled MOFs is highly desired, as well as the exploration of their potential applications.

**Scheme 1:**
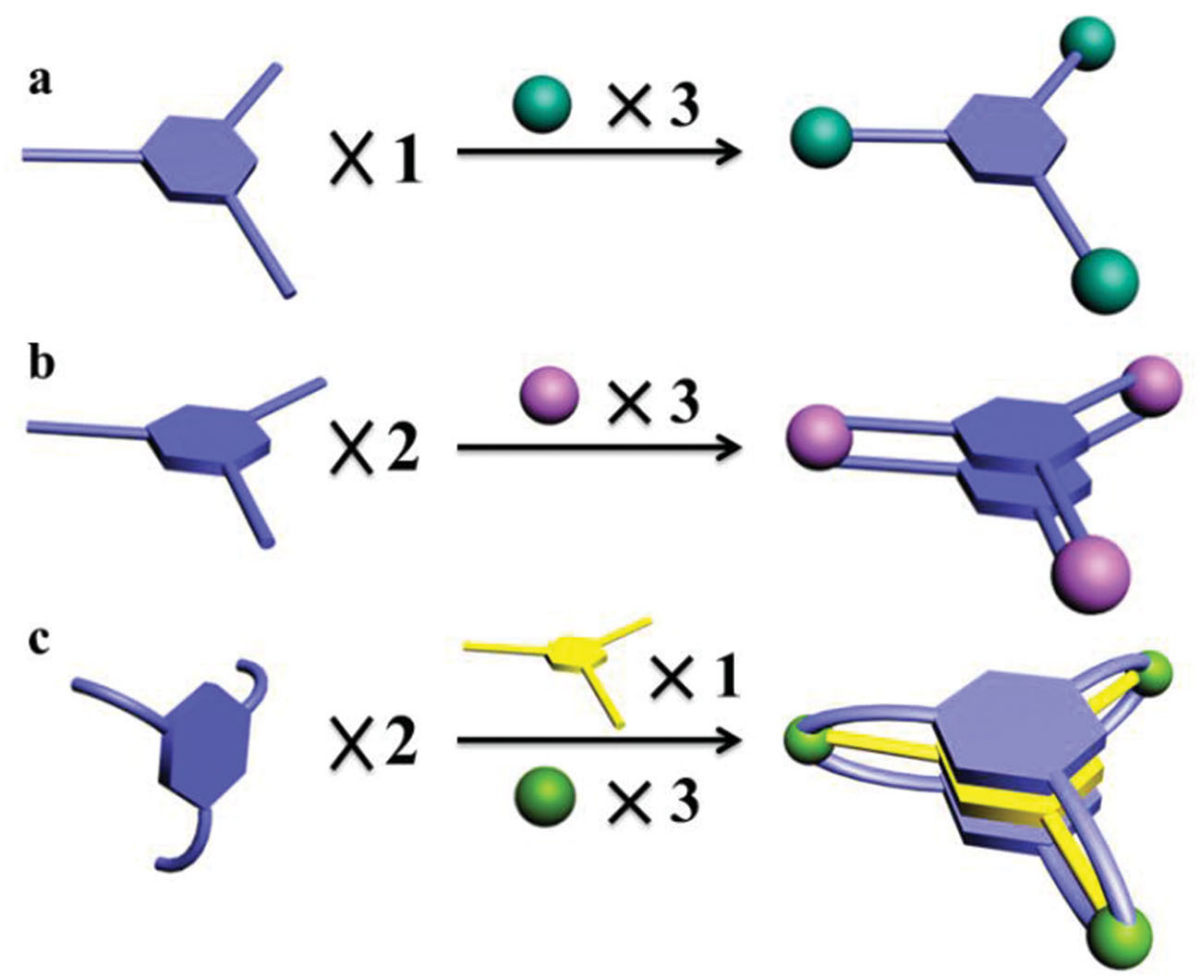
Schematic representation of the rational design of single‐, double‐, and triple‐layered building blocks based on *C*_3_‐symmetric trigonal MBBs.

Conceptually, the multiwalled MOF frameworks can be built up from the assembly of multilayered building blocks with metal centers or clusters as vertices. In general, such multilayered configuration usually requires both the shape‐ and size‐matching between individual layers. Thus, the simplest way to simultaneously meet these two requirements is to build up a parallel stacking of multi‐identical MBBs. In line with this principle, several double‐walled MOFs have been reported recently, the constituent double‐layered building blocks of which are primarily constructed with two identical linear^25^, ^26^, ^30^, ^31^, ^32^, ^33^ or trigonal^34^, ^35^ rigid MBBs (e.g., Scheme 1b). This strategy is in principle also applicable to design higher‐level multiwalled MOFs, but, to the best of our knowledge, only Rosi's group reported two 3D porous MOFs exhibiting triple‐walled frameworks.^27^, ^28^ Therein, a triplex linear rigid linker was adopted to form the triple‐layered building block with a rare metal‐biomolecule cluster of an apparently large size employed as vertex due to the stringent steric constraint. These examples have revealed two fundamental limitations of this multiwalled MOF design strategy. First, the size requirement of the metal‐cluster vertices would increase considerably with the number of walls (i.e., the thickness of framework wall), which may cause serious difficulties in the target design and synthesis. Second, the resulting multiple framework walls are of the same size, shape, and even rigidity. This thus leads to some degree of loss of compositional diversity that may impact the functional versatility of multiwalled MOFs.

Alternatively, the rational design of multilayered building block can be implemented in a different manner, by assigning the shape‐matching MBBs of different sizes and rigidities as individual layers. In this way, the longer, flexible organic ligands are exploited because of their considerable tunability in geometry and tend to form the exterior layers of target composite building block, while the shorter, rigid linkers can be encapsulated between them to serve as the interior layers for stabilizing the framework. It is worth mentioning that this strategy of integrating individual flexible and rigid MBBs into one composite building block, called the mixed MBB strategy,^36^ was proposed and applied to achieve two isostructural nested polyhedron MOFs (a representative form of double‐walled MOFs with cage‐in‐cage structure) in our recent study.^36^ Likewise, it is then anticipated to extend this strategy to fabricate the higher‐level multiwalled architectures, whereby it can theoretically provide more diversity in terms of different combinations of MBBs, e.g., one of the possible configurations for a hetero‐triple‐layered building block is illustrated in Scheme 1c.

Intrinsically, the mixed MBB strategy can easily address the two aforementioned limitations in developing multiwalled MOFs with the strategy based on multi‐identical rigid MBBs. First, the mixed MBB strategy promises a high degree of structural flexibility due to the participation of flexible organic ligands. The tunable geometries of these involved flexible MBBs could not only facilitate the size‐matching requirement by adapting their lengths and configurations upon conformational alternation, but could also effectively reduce the steric hindrance between adjacent MBBs, and thus the size penalty for metal‐cluster vertices. This then makes it possible to employ a conventional metal‐carboxylate cluster of relatively small size as vertex to gather and dispose multiple MBBs, thus enabling the commonly used carboxylate‐linker approach in the design of multiwalled MOFs. Second, the rigidity of individual framework wall is determined by its constituent MBB, which thus leads to a significant diversity in the character of the resulting framework walls, i.e., those featuring different compositions and rigidities. This unique structural characteristic may engender new functions and/or significantly improve the inherent properties of MOF materials.

By means of the afore‐described mixed MBB strategy, we successfully synthesized, to our knowledge, the first 3D hetero‐triple‐walled MOFs known to date, formulated as {[Co_6_L_4_(TPT)_2_(*μ*_3_‐OH)_2_]·Co(H_2_O)_6_·xG}_n_ (**1**) (TPT = 2,4,6‐tris(4‐pyridyl)‐1,3,5‐triazine, H_3_L = 2,4,6‐tris[1‐(3‐carboxylphenoxy)ylmethyl]mesitylene, G = guest molecules). Here, two trigonal planar ligands of different sizes and rigidities were employed, namely TPT and H_3_L, bearing electron‐deficient triazine ring and electron‐rich benzene ring, respectively. Evidently, such a shape complementarity between ligands involving mutually attractive species will largely facilitate the integration of multiple MBBs. Then, the well‐known trigonal prismatic secondary building unit (SBU), [M_3_(μ_3_‐O)(O_2_CR)_6_], is selected so as to coincide with the *C*_3_‐symmetry of ligands, which consists of octahedrally coordinated metal ions and is more beneficial to form the highly symmetrical structures.^37^ During a face‐directed self‐assembly process, two flexible H_3_L ligands and one rigid TPT ligand are “pinched together” at three trinuclear cobalt‐carboxylate SBUs, Co_3_O(COO)_6_, with their central parts packed in parallel to form a “sandwich‐like” hetero‐triple‐layered building block (**Figure** **1**a), denoted hereafter as L^3−^‐TPT‐L^3−^, for clarity. While the geometry of SBUs determines the topology of the resulting framework, the mixed composition of MBBs with different sizes and rigidities in multilayered building blocks will likely influence some specific properties of **1**.

**Figure 1:**
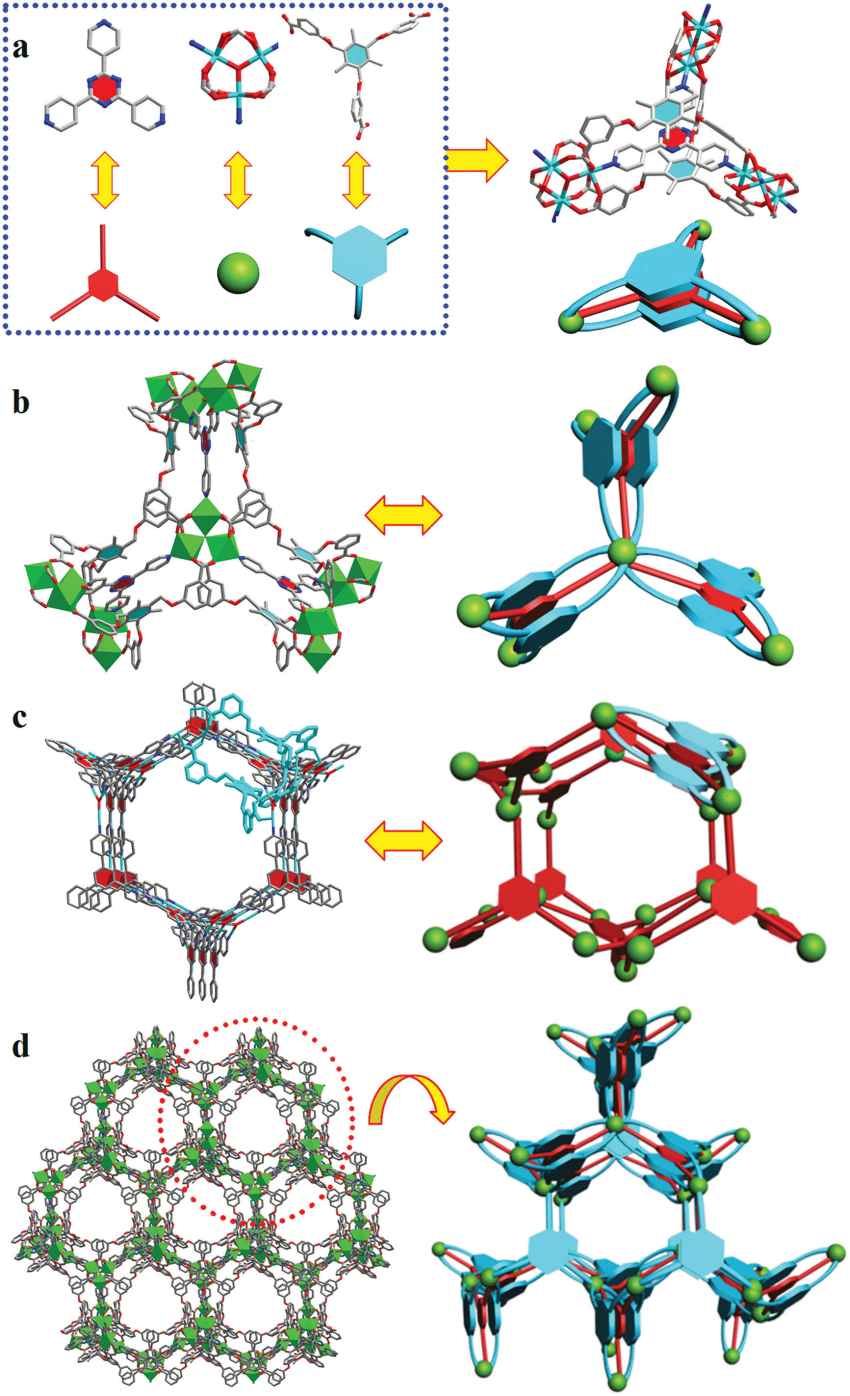
a) Sandwich‐like hetero‐triple‐layered building block assembled with three trinuclear Co_3_ SBUs and three *C*_3_‐symmetric MBBs of different sizes and rigidities; Views of b) a propeller arrangement at Co_3_ SBU; c) the 1D hexagonal channel; and d) the 3D framework along [111].

Complex **1** was obtained by the solvothermal reaction of H_3_L, TPT, and Co(NO_3_)_2_·6H_2_O in DMF/C_2_H_5_OH/H_2_O at 95 °C for 72 h. Single‐crystal structural analysis reveals that **1** crystallizes in the cubic space group *Pa‐*3 and possesses a 3D coordination network consisting of the hetero‐triple‐layered building blocks interconnected via the trinuclear Co_3_ SBUs. In **1**, there exist two crystallographically independent Co^2+^ ions (Figure S1, Supporting Information), denoted as Co1^2+^ and Co2^2+^, respectively. Each Co1^2+^ ion is surrounded by four carboxylate oxygen atoms in equatorial positions, as well as one μ_3_‐O13 atom and one pyridine nitrogen atom in axial positions, thus exhibiting a slightly distorted octahedral geometry. Based on this coordination configuration, three equivalent Co1^2+^ ions (Co···Co: 3.488 Å) are linked together by six carboxylate groups from six individual L^3−^ ligands and one μ_3_‐O13 atom to form a trinuclear Co_3_O(COO)_6_ SBU, with all the Co—O distances falling in the range of 1.98(3)–2.23(2) Å. On the other hand, each Co2^2+^ ion is coordinated by six water molecules and lies right above the trinuclear Co_3_ SBU with the distance of about 5.717 Å between Co2 and O13 (Figure S1a, Supporting Information).

Each hetero‐triple‐layered building block (L^3−^‐TPT‐L^3−^; Figure 1a) involves three neighboring Co_3_O(COO)_6_ SBUs as vertices that are arranged in an equilateral triangle in accordance with the trigonal symmetry of both L^3−^ and TPT ligands (Figure 1a). In this unit, each flexible L^3−^ ligand is coordinated by six Co1^2+^ ions from three individual Co_3_ SBUs, while each rigid TPT ligand coordinates only to the inner three Co1^2+^ ions and serves as the interior layer between the two exterior L^3−^ layers. For each L^3−^ ligand, its central benzene ring and peripheral flexible phenyl carboxyl groups are linked by —CH_2_—O— groups. Through the rotation of this single bond, the geometry of L^3−^ can be tuned to adjust the distance between its end *meta* carboxyl groups to match with the size of TPT, in accompany with the distortion of the peripheral phenyl moieties that are preferentially perpendicular to the central benzene ring (Figure S1b, Supporting Information).

Close examination of the formed L^3−^‐TPT‐L^3−^ composite structure reveals that the two involved L^3−^ MBBs show the same *syn*‐*syn*‐*syn* configuration (Figure S1b, Supporting Information) and are mirror located at the two sides of one TPT ligand with their central parts arranged in parallel, thus resembling a compressed chamber with high symmetry (Figure S1c, Supporting Information). The spontaneously adopted parallel stacking of the “sandwich‐like” triple layers indicates the presence of a weak π···π interaction (3.61–4.01 Å) between the central electron‐deficient triazine and electron‐rich benzene ring, from adjacent TPT and L^3−^ ligands, respectively (Figure S1d, Supporting Information).

From another point of view, each Co_3_ SBU connects with three L^3−^‐TPT‐L^3−^ pairs to form a left‐ or right‐handed chiral propeller‐type unit (Figure 1b; and S2a, Supporting Information). Upon this connectivity, the formed propellers can propagate the chirality throughout the whole triple‐walled network, including the inside 1D channels of ≈11 Å in diameter (without considering van der Waals radii; Figure 1c). The 3D packing of the chiral propeller units by sharing the same Co_3_ SBU tends to produce a chiral open framework (Figure 1d and S2b, Supporting Information), which can be topologically classified as a srs network (Figure S3b, Supporting Information) with the Schläfli symbol of (10^3^) when considering both L^3−^‐TPT‐L^3−^ and Co_3_O(COO)_6_ as 3‐connected nodes (Figure S3a, Supporting Information). Moreover, each of the resulting chiral 3D networks, either left‐ or right‐handed, involves two different types of helix chains (left‐ versus right‐handed) with the same pitch length of ≈27 Å (Figure S4, Supporting Information). Through self‐assembly of these 3D chiral nets, the first hetero‐triple‐walled MOF (**1**) is then formed but found to be racemic due to the interpenetration of the chiral nets of opposite handedness (Figure S5, Supporting Information).

It is worth noting that both the TPT and H_3_L ligands were used in our previous study to construct the cage‐in‐cage double‐walled MOFs, [M_3_L_2_(TPT)_2_·xG]*_n_* (M = Ni or Co),^36^ constituting the inner and outer cages, respectively. Therein, each paddle‐wheel SBU, Co_2_(CO_2_)_4_, serves as the common vertex for two hetero‐double‐layered building blocks (L^3−^‐TPT), bearing in mind that in **1**, each trinuclear Co_3_O(COO)_6_ SBU gathers three L^3−^‐TPT‐L^3−^ pairs. In comparison, it is observed that every two TPT ligands at the same SBU are non‐coplanar, for either Co_2_(CO_2_)_4_ or Co_3_O(COO)_6_, but associated with different dihedral angles between the two central triazine rings (70.54° versus 110.55°; Figure S6, Supporting Information). The significant difference in dihedral angles consequently leads to two very distinct multi­walled architectures: the L^3−^ and TPT MBBs linked through Co_2_(CO_2_)_4_ SBUs adapt to form a closed polyhedral structure, while those at Co_3_O(COO)_6_ SBUs tend to produce an open network structure (**Figure** **2**). These results thus indicate that the geometry of SBUs directs the assembly of MBBs and determines the topology of ordered networks.^38^ More significantly, the methodological consistency in fabricating both the double‐walled and triple‐walled MOFs with similar composition can be regarded as a manifestation of the generality and the robustness of the mixed MBB strategy, which in a sense, also suggests a great potential in achieving an even greater level of multiwalled architectures, once the appropriate SBUs were identified.

**Figure 2:**
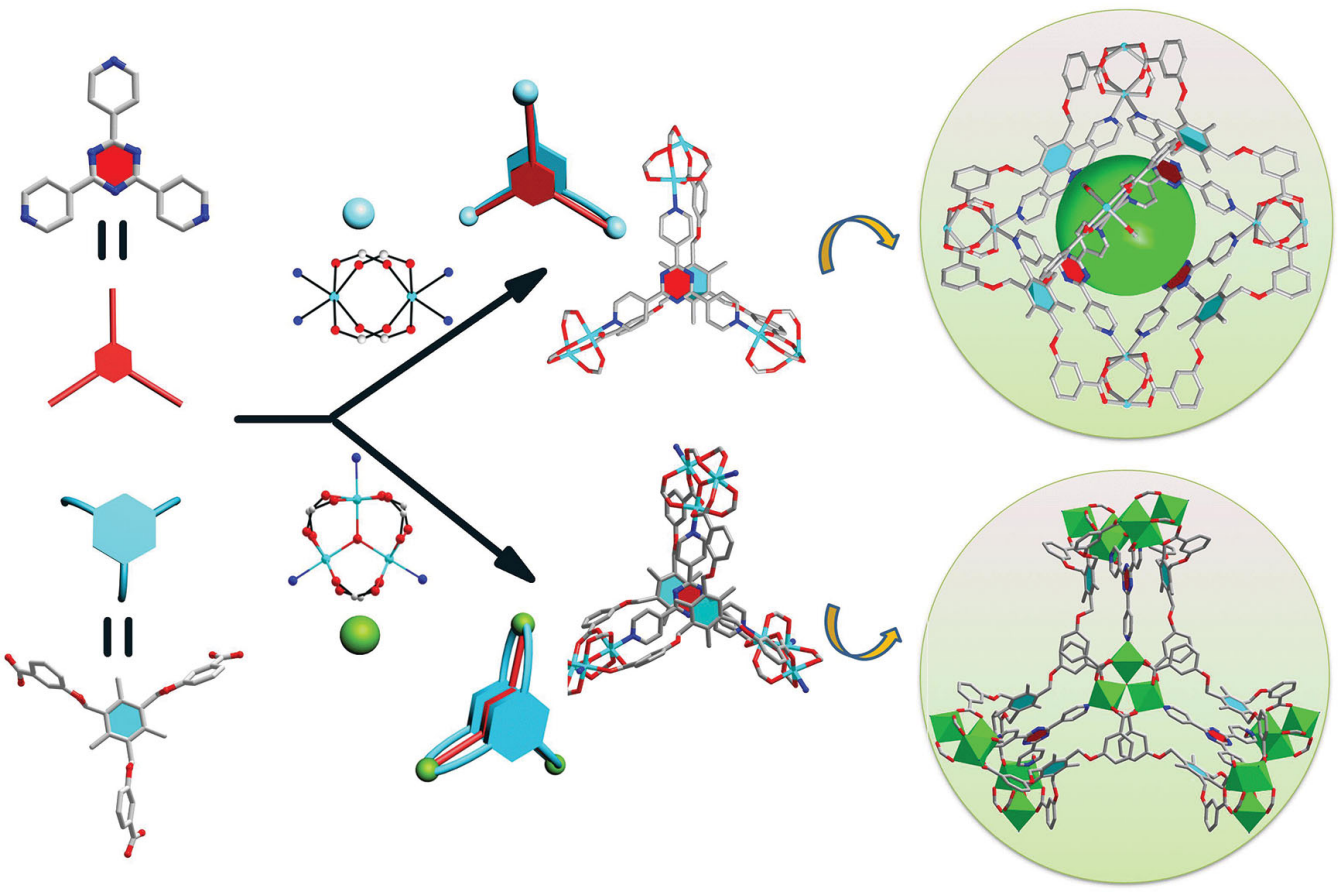
The rational designs of double‐layered and triple‐layered composite building blocks.

The aesthetically pleasing multiwalled architecture is a unique structural attribute that may result in a high degree of structural robustness.^29^ Remarkably, the powder X‐ray diffraction (PXRD) patterns reveal that **1** retained its crystallinity after immersed in water for more than 7 d, even in boiling water for at least 48 h (**Figure** **3**), thus exhibiting an exceptional water‐resistance ability that is of great concern in the field of MOF materials.^39^, ^40^, ^41^ Further investigations indicate that the sample of **1** could also retain its crystallinity in aqueous solutions associated with a broad pH value range of 2–9 (Figure 3), as well as in many common organic solvents, such as methanol, acetone, tetrahydrofuran, acetonitrile, dichloromethane, and trichloromethane (Figure S8, Supporting Information). It is worth noting that the slight PXRD peak shifts in the low‐angle region might be due to the solvent influence.^42^, ^43^, ^44^ All these results strongly suggest good feasibility of **1** in a broad variety of applications, even under complex working conditions. In addition, the thermal stability of **1** was also investigated by conducting thermogravimetric analysis (TGA) and variable‐temperature PXRD measurements. The TGA profiles reveal that the framework of **1** is thermally stable below 350 °C (Figure S9, Supporting Information), and that a weight loss corresponding to the removal of encapsulated solvents occurs in accompany with a interpenetrated net sliding phenomenon, which also results in the slight peak shifts in the low‐angle region of the variable temperature PXRD patterns (Figure S10, Supporting Information).^42^, ^43^, ^44^ Upon activation at 110 °C, the encapsulated solvent molecules can be completely removed to yield the guest‐free sample of **1**, as confirmed by the TGA profiles shown in Figure S11 (Supporting Information), thus indicating that the hetero‐triple‐walled framework of **1** is structurally robust upon removal of encapsulated solvent molecules. In order to justify the influence of multiwalled organization on the framework stability, we also compared the chemical stability of the triple‐walled MOF with its double‐walled ([Co_3_L_2_(TPT)_2_·xG]*_n_*)^36^ and single‐walled [(Co(SCN)_2_)_3_(TPT)_4_·xG]*_n_* 45 counterparts. The results indicate that both the double‐walled and single‐walled MOFs exhibit the structural change in water, evidenced by the prominent reduction in intensities (Figure S12, Supporting Information) and considerable peak shifts of the PXRD patterns (Figure S13, Supporting Information). As known, there are also many other factors that affect the framework stability, such as interpenetration, porosity and other discrepancies in structure, we therefore coule not assign the remarkable chemical stability of the title MOF exclusively to its hetero‐triple‐walled framework. But in a sense, this comparison indeed implies constructing the multiwalled framework may be a promising way to improve the chemical stability of MOF materials and deserves to be further explored from both theoretical and experimental perspectives.

**Figure 3:**
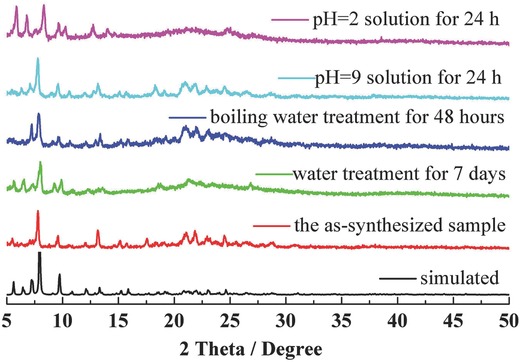
PXRD profiles for simulated, as‐synthesized **1**, and **1** soaked in water, boiling water and aqueous solutions with pH values of 2 and 9.

Recently, there has been growing interest in developing MOFs as a new class of electrode materials for Li‐ion batteries (LIBs),^46^, ^47^, ^48^, ^49^, ^50^, ^51^ in which case high degrees of structural flexibility and chemical stability are favored. While feasible structural tunability can assist in the breathing of electrode materials and thereby enhance the cyclability of LIBs,^47^ high structural stability at electrochemical conditions may prevent the irreversible destruction of the MOF framework.^48^ Thus, we then proceeded with the exploration of the Li storage performance of **1** as anode material. Figure S14 (Supporting Information) displays the representative charge–discharge curves for various cycles at a current density of 50 mA g^−1^ in potential range between 0.01 and 3.00 V. During the initial cycle, moderate specific discharge and charge capacities of ≈1108 and 345 mAh g^−1^ were achieved. The large irreversible capacity observed here is probably due to inevitable formation of solid electrolyte interface (SEI) film and extended electrolyte degradation,^52^, ^53^ both of which are very common for most anode materials of LIBs.

Detailed analysis of the cyclic voltammograms (CVs) was also conducted to examine the electrochemical behavior of **1**. As seen from the inset of **Figure** **4**, the initial cathodic scan reveals three main peaks at 1.42, 1.00, and 0.65 V, respectively. While the peaks at 1.42 and 1.00 V correspond to the amorphization and the reduction of Co ions,^47^ the intense cathodic peak at 0.65 V and broad peak below 0.5 V are ascribed to the formation of SEI film and the electrolyte decomposition.^52^, ^53^ Moreover, the initial anodic scan also exhibits two hump‐like peaks at 1.02 and 1.25 V, which are indicative of the oxidation and reformation of **1**.^47^ Evidently, the subsequent CV curves differ significantly from the initial one and show very good reproducibility in peak shape, thus demonstrating a good reversibility in Li storage. Thus, the cyclic performance of **1** indicates that, despite a decline of reversible capacity in the initial several cycles, a nearly invariable capacity of about 300 mAh g^−1^ can be obtained in the following cycles (Figure 4), with the coulombic efficiency of >95% after 15 cycles. A similar situation also occurred at a current density of 100 mA g^−1^, exhibiting a reversible capacity of about 160 mAh g^−1^ (Figure S15, Supporting Information).

**Figure 4:**
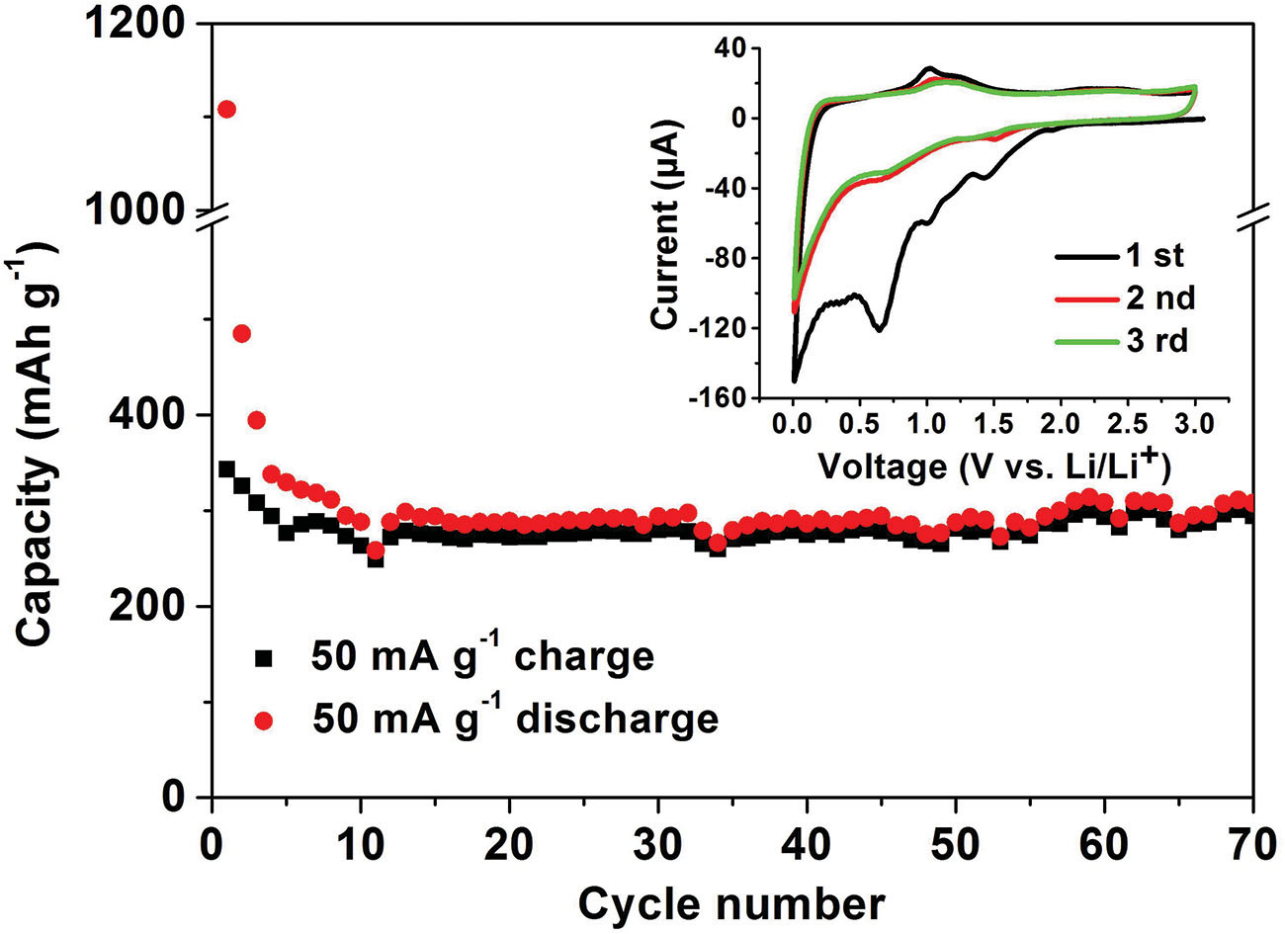
Cycling performance of **1** at a current density of 50 mA g^−1^. (inset) CV curves of 1–3 cycles in the potential range of 0.01–3.00 V vs. Li/Li^+^ at a scan rate of 0.1 mV s^−1^.

The results presented above reveal that **1** exhibits a superior or comparable Li storage performance, especially in cyclability, as compared with many developed MOF electrodes,^49^, ^50^, ^51^ and even inorganic TiO_2_ material.^54^, ^55^, ^56^ More significantly, in sharp contrast to the reversible Li storage dominated by the derived metal‐oxides or metal nanoalloys as in many MOF‐based LIBs, the matrix involved in this test almost retained during the cycling as evidenced by the unchanged XRD patterns (Figure S16, Supporting Information). Therein, the hetero‐triple‐walled framework of **1** not only helps to avoid the capacity decay and thus improve the cyclability owing to its high chemical stability, but also provides more redox‐active sites from multiple ligands within a confined pore space,^57^, ^58^ displaying a selectivity to Li and thereby preventing the entering of other irrelevant materials for protection. Since to date, only a few of MOF electrodes can survive or regenerate during the cycles,^46^, ^47^, ^48^ we then suggest that manufacturing the multiwalled MOF electrodes may be a promising way to improve the cyclic performance of LIBs.

In summary, we elaborate an alternative method, i.e., the mixed MBB strategy, for constructing the multiwalled MOFs of high structural complexity and aesthetic appeal, under which the shape‐matching flexible and rigid MBBs are integrated into one composite building block as separate layers. Compared to the strategy based on multi‐identical rigid MBBs, this new strategy not only enables more compositional diversity, especially in the character of framework walls, but also promises higher structural flexibility that could effectively reduce the dependence of multilayered building block on the size of metal‐cluster vertices. With the methodological development in engineering of multiwalled MOFs, we obtained an unprecedented 3D hetero‐triple‐walled MOF with remarkable structural flexibility and chemical stability, which could significantly improve the cyclability of MOF materials in the application of LIB electrodes. Therefore, we anticipate that the proposed mixed MBB strategy could not only offer a new platform for developing novel MOF structures, but also provide new insights into the functionalization of this class of crystalline materials.

## Supporting information

As a service to our authors and readers, this journal provides supporting information supplied by the authors. Such materials are peer reviewed and may be re‐organized for online delivery, but are not copy‐edited or typeset. Technical support issues arising from supporting information (other than missing files) should be addressed to the authors.

Supplementary

## References

[1] J. J. Perry IV , J. A. Perman , M. J. Zaworotko , Chem. Soc. Rev. 2009, 38, 1400. 19384444 10.1039/b807086p

[2] D. J. Tranchemontagne , Z. Ni , M. O'Keeffe , O. M. Yaghi , Angew. Chem., Int. Ed. 2008, 47, 5136. 10.1002/anie.200705008 18528833

[3] S. R. Seidel , P. J. Stang , Acc. Chem. Res. 2002, 35, 972. 12437322 10.1021/ar010142d

[4] Y. B. He , B. Li , M. O'Keeffe , B. L. Chen , Chem. Soc. Rev. 2014, 43, 5618. 24705653 10.1039/c4cs00041b

[5] J. P. Zhang , Y. B. Zhang , J. B. Lin , X. M. Chen , Chem. Rev. 2012, 112, 1001. 21939178 10.1021/cr200139g

[6] D. S. Zhang , Z. Chang , Y. F. Li , Z. Y. Jiang , Z. H. Xuan , Y. H. Zhang , J. R. Li , Q. Chen , T. L. Hu , X. H. Bu , Sci. Rep. 2013, 3, 3312. 24264725 10.1038/srep03312 PMC3837364

[7] H. R. Fu , Z. X. Xu , J. Zhang , Chem. Mater. 2015, 27, 205.

[8] C. Y. Sun , X. L. Wang , X. Zhang , C. Qin , P. Li , Z. M. Su , D. X. Zhu , G. G. Shan , K. Z. Shao , H. Wu , J. Li , Nat. Commun. 2013, 4, 2717. 24212250 10.1038/ncomms3717 PMC3831296

[9] S. Tominaka , H. Hamoudi , T. D. Bennett , A. B. Cairns , A. K. Cheetham , Chem. Sci. 2015, 6, 1465. 10.1039/c4sc03295k PMC5811114 29560235

[10] Z. Y. Du , T. T. Xu , B. Huang , Y. J. Su , W. Xue , C. T. He , W. X. Zhang , X. M. Chen , Angew. Chem., Int. Ed. 2015, 54, 914. 10.1002/anie.201408491 25430633

[11] S. T. Zheng , J. T. Bu , Y. F. Li , T. Wu , F. Zuo , P. Y. Feng , X. H. Bu , J. Am. Chem. Soc. 2010, 132, 17062. 21080641 10.1021/ja106903p

[12] S. T. Zheng , T. Wu , B. Irfanoglu , F. Zuo , P. Y. Feng , X. H. Bu , Angew. Chem., Int. Ed. 2011, 50, 8034. 10.1002/anie.201103155 21761532

[13] J. Park , D. W. Feng , H. C. Zhou , J. Am. Chem. Soc. 2015, 137, 1663. 25581395 10.1021/ja5123528

[14] O. K. Farha , A. O. Yazaydın , I. Eryazici , C. D. Malliakas , B. G. Hauser , M. G. Kanatzidis , S. T. Nguyen , R. Q. Snurr , J. T. Hupp , Nat. Chem. 2010, 2, 944. 20966950 10.1038/nchem.834

[15] Y. W. Li , K. H. He , X. H. Bu , J. Mater. Chem. A 2013, 1, 4186.

[16] S. S. Mondal , A. Bhunia , A. Kelling , U. Schilde , C. Janiak , H. Holdt , J. Am. Chem. Soc. 2014, 136, 44. 24313724 10.1021/ja410595q

[17] H. X. Deng , C. J. Doonan , H. Furukawa , R. B. Ferreira , J. Towne , C. B. Knobler , B. Wang , O. M. Yaghi , Science 2010, 327, 846. 20150497 10.1126/science.1181761

[18] L. J. Liu , K. Konstas , M. R. Hill , S. G. Telfer , J. Am. Chem. Soc. 2013, 135, 17731. 24180695 10.1021/ja4100244

[19] J. Zou , B. H. Ji , X. Q. Feng , H. J. Gao , Nano Lett. 2006, 6, 430. 16522036 10.1021/nl052289u

[20] W. S. Su , T. C. Leung , C. T. Chan , Phys. Rev. B 2007, 76, 235413.

[21] S. S. Y. Chui , S. M. F. Lo , J. P. H. Charmant , A. G. Orpen , I. D. Williams , Science 1999, 283, 1148. 10024237 10.1126/science.283.5405.1148

[22] M. Dincă , W. S. Han , Y. Liu , A. Dailly , C. M. Brown , J. R. Long , Angew. Chem., Int. Ed. 2007, 46, 1419. 10.1002/anie.200604362 17387653

[23] F. J. Ma , S. X. Liu , C. Y. Sun , D. D. Liang , G. J. Ren , F. Wei , Y. G. Chen , Z. M. Su , J. Am. Chem. Soc. 2011, 133, 4178. 21370859 10.1021/ja109659k

[24] S. Q. Ma , H. C. Zhou , J. Am. Chem. Soc. 2006, 128, 11734. 16953594 10.1021/ja063538z

[25] W. Y. Gao , W. M. Yan , R. Cai , L. Meng , A. Salas , X. S. Wang , L. Wojtas , X. D. Shi , S. Q. Ma , Inorg. Chem. 2012, 51, 4423. 22449128 10.1021/ic3002256

[26] J. Seo , H. Chun , Eur. J. Inorg. Chem. 2009, 4946.

[27] J. An , S. J. Geib , N. L. Rosi , J. Am. Chem. Soc. 2009, 131, 8376. 19489551 10.1021/ja902972w

[28] J. An , O. K. Farha , J. T. Hupp , E. Pohl , J. I. Yeh , N. L. Rosi , Nat. Commun. 2012, 3, 604. 22215079 10.1038/ncomms1618

[29] K. J. Chen , J. J. Perry IV , H. S. Scott , Q. Y. Yang , M. J. Zaworotko , Chem. Sci. 2015, 6, 4784. 10.1039/c5sc01515d PMC5502393 28717485

[30] M. H. Zeng , Q. X. Wang , Y. X. Tan , S. Hu , H. X. Zhao , L. S. Long , M. Kurmoo , J. Am. Chem. Soc. 2010, 132, 2561. 20131766 10.1021/ja908293n

[31] Z. B. Han , R. Y. Lu , Y. F. Liang , Y. L. Zhou , Q. Chen , M. H. Zeng , Inorg. Chem. 2012, 51, 674. 22141338 10.1021/ic2021929

[32] D. T. Vodak , M. E. Braun , J. Kim , M. Eddaoudi , O. M. Yaghi , Chem. Commun. 2001, 2534.

[33] Z. Z. Lu , R. Zhang , Z. R. Pan , Y. Z. Li , Z. J. Guo , H. G. Zheng , Chem. Eur. J. 2012, 18, 2812. 22307561 10.1002/chem.201101963

[34] Z. R. Pan , J. Xu , H. G. Zheng , K. X. Huang , Y. Z. Li , Z. J. Guo , S. R. Batten , Inorg. Chem. 2009, 48, 5772. 19499896 10.1021/ic802457j

[35] D. F. Sun , Y. X. Ke , D. J. Collins , G. A. Lorigan , H. C. Zhou , Inorg. Chem. 2007, 46, 2725. 17348646 10.1021/ic0624773

[36] D. Tian , Q. Chen , Y. Li , Y. H. Zhang , Z. Chang , X. H. Bu , Angew. Chem., Int. Ed. 2014, 53, 837. 10.1002/anie.201307681 24282117

[37] A. Schoedel , M. J. Zaworotko , Chem. Sci. 2014, 5, 1269.

[38] M. Köberl , M. Cokoja , W. A. Herrmann , F. E. Kühn , Dalton Trans. 2011, 40, 6834. 21416068 10.1039/c0dt01722a

[39] S. S. Nagarkar , A. K. Chaudhari , S. K. Ghosh , Inorg. Chem. 2012, 51, 572. 22148730 10.1021/ic202102m

[40] A. Demessence , D. M. DAlessandro , M. L. Foo , J. R. Long , J. Am. Chem. Soc. 2009, 131, 8784. 19505094 10.1021/ja903411w

[41] H. Jasuja , N. C. Burtch , Y. G. Huang , Y. Cai , K. S. Walton , Langmuir 2013, 29, 633. 23214448 10.1021/la304204k

[42] A. Schneemann , V. Bon , I. Schwedler , I. Senkovska , S. Kaskel , R. A. Fischer , Chem. Soc. Rev. 2014, 43, 6062. 24875583 10.1039/c4cs00101j

[43] Y. Sakata , S. Furukawa , M. Kondo , K. Hirai , N. Horike , Y. Takashima , H. Uehara , N. Louvain , M. Meilikhov , T. Tsuruoka , S. Isoda , W. Kosaka , O. Sakata , S. Kitagawa , Science 2013, 339, 193. 23307740 10.1126/science.1231451

[44] J. Seo , C. Bonneau , R. Matsuda , M. Takata , S. Kitagawa , J. Am. Chem. Soc. 2011, 133, 9005. 21553824 10.1021/ja201484s

[45] S. Matsuzaki , T. Arai , K. Ikemoto , Y. Inokuma , M. Fujita , J. Am. Chem. Soc. 2014, 136, 17899. 25495652 10.1021/ja5109535

[46] G. Férey , F. Millange , M. Morcrette , C. Serre , M. L. Doublet , J. M. Greneche , J. M. Tarascon , Angew. Chem., Int. Ed. 2007, 46, 3259. 10.1002/anie.200605163 17385766

[47] K. Saravanan , M. Nagarathinam , P. Balaya , J. J. Vittal , J. Mater. Chem. 2010, 20, 8329.

[48] Y. C. Lin , Q. J. Zhang , C. C. Zhao , H. L. Li , C. L. Kong , C. Shen , L. Chen , Chem. Commun. 2015, 51, 697. 10.1039/c4cc07149b 25418209

[49] Q. Liu , L. L. Yu , Y. Wang , Y. Z. Ji , J. Horvat , M. L. Cheng , X. Y. Jia , G. X. Wang , Inorg. Chem. 2013, 52, 2817. 23461562 10.1021/ic301579g

[50] L. Gou , L. M. Hao , Y. X. Shi , S. L. Ma , X. Y. Fan , L. Xu , D. L. Li , K. Wang , J. Solid State Chem. 2014, 210, 121.

[51] X. X. Li , F. Y. Cheng , S. N. Zhang , J. Chen , J. Power Sources 2006, 160, 542.

[52] L. W. Su , Y. R. Zhong , Z. Zhou , J. Mater. Chem. A 2013, 1, 15158.

[53] Y. R. Zhong , L. W. Su , M. Yang , J. P. Wei , Z. Zhou , ACS Appl. Mater. Interfaces 2013, 5, 11212. 24066809 10.1021/am403453r

[54] G. Armstrong , A. R. Armstrong , J. Canales , P. G. Bruce , Chem. Commun. 2005, 2454. 10.1039/b501883h 15886768

[55] J. S. Chen , X. W. Lou , J. Power Sources 2010, 195, 2905.

[56] M. M. Zhen , X. J. Guo , G. D. Gao , Z. Zhou , L. Liu , Chem. Commun. 2014, 50, 11915. 10.1039/c4cc05480f 25156333

[57] X. Han , G. Qing , J. Sun , T. Sun , Angew. Chem., Int. Ed. 2012, 51, 5147. 10.1002/anie.201109187 22511505

[58] J. Wu , X. Rui , G. Long , W. Chen , Q. Yan , Q. Zhang , Angew. Chem., Int. Ed. 2015, 54, 7354. 10.1002/anie.201503072 25960289

