# Supplementary material for: The First Example of Hetero‐Triple‐Walled Metal–Organic Frameworks with High Chemical Stability Constructed via Flexible Integration of Mixed Molecular Building Blocks
Source: Adv Sci (Weinh). 2015 Dec 3;3(10):1500283. doi: 10.1002/advs.201500283 (PMC5095769; doi:10.1002/advs.201500283)
Supplement: Supplementary file 1 — Supplementary [file ADVS-3-0p-s001.pdf]

Copyright WILEY-VCH Verlag GmbH & Co. KGaA, 69469 Weinheim, Germany, 2015.

ADVANCED SCIENCE
Open Access

Supporting Information

for *Adv. Sci.*, DOI: 10.1002/advs.201500283

The First Example of Hetero-Triple-Walled Metal–Organic Frameworks with High Chemical Stability Constructed via Flexible Integration of Mixed Molecular Building Blocks

*Dan Tian, Jian Xu, Zhao-Jun Xie, Zhao-Quan Yao, Deng-Lin Fu, Zhen Zhou, and Xian-He Bu**

Supporting Information

The first example of hetero-triple-walled metal-organic frameworks with high chemical stability constructed via flexible integration of mixed molecular building blocks

Dan Tian, Jian Xu, Zhao-Jun Xie, Zhao-Quan Yao, Deng-Lin Fu, Zhen Zhou and Xian-He Bu*

Department of Chemistry, TKL of Metal- and Molecule-based Material Chemistry, Nankai University, Tianjin 300071, China.

Collaborative Innovation Center of Chemical Science and Engineering (Tianjin).

*Corresponding author: Xian-He Bu. (email: buxh@nankai.edu.cn)

Materials and syntheses.

All the chemicals used for synthesis are of analytical grade and commercially available. IR spectra were measured on a Tensor 27 OPUS (Bruker) FT-IR spectrometer with KBr pellets. Powder X-ray diffraction (PXRD) spectra were recorded on a Rigaku D/Max-2500 diffractometer at 40 kV, 100 mA for a Cu-target tube and a graphite monochromator. The variable-temperature X-ray powder diffraction was recorded with a Panalytical X'pert PRO diffractometer at 40 kV, 30 mA with a Cu-target tube and a graphite monochromator. Thermogravimetric analyses (TGA) were carried out on a Rigaku standard TG-DTA analyzer under N_2 with a heating rate of 10 °C min^{-1}, with an empty Al_2O_3 crucible used as reference. Simulation of the PXRD pattern was carried out by the single-crystal data and diffraction-crystal module of the Mercury (Hg) program version 1.4.2 available free of charge via the Internet at http://www.iucr.org. Simplification of the network and its topological analysis are readily achieved using the program TOPOS.[S1]

X-ray single-crystal diffraction.

Single crystal X-ray diffraction measurement was carried out on Rigaku Saturn70 diffractometer at 113(2) K for **1** with Mo-*Kα* radiation (λ = 0.71073 Å). The structure was solved by direct methods using the SHELXS program of the SHELXTL package and refined with SHELXL.[S2] It should be noted that the guest molecules in **1** are highly disordered and could not be modelled properly, so the diffused electron densities resulting from them were removed by the SQUEEZE routine in PLATON[S3] and the result was appended in the CIF file. Crystal and refinement information: complex **1**: cubic, *P*a-3, a = 27.391(3) Å, V = 20551(4) $Å^3$, T = 113(2) K, Z = 4, R_1 = 0.1342, wR_2 = 0.3826. CCDC 1047575 contains the supplementary crystallographic data for this paper. These data can be obtained free of charge from The Cambridge Crystallographic Data Centre via www.ccdc.cam.ac.uk/data_request/cif.

Synthesis of {[$Co_6L_4(TPT)_2(\mu_3\text{-}OH)_2$]·$Co(H_2O)_6$·xG}$_n$ (1)

A mixture of $Co(NO_3)_2\cdot 6H_2O$ (0.070 mmol), H_3L (0.035 mmol), TPT (0.035 mmol) and 6 mL DMF/C_2H_5OH/H_2O (v/v/v = 3:2:1) was sealed in a 10 ml vial and heated to 95 °C for 72 hours. The reaction vessel was cooled to room temperature. The red crystals of **1** were collected by filtration, followed by several washings with DMF and ethanol to remove the attached metal ions and ligands. Finally, the sample (yield: ca 45% based on H_3L) was dried overnight at room temperature. FT-IR (KBr pellets, cm^{-1}):

3381s, 3068m, 2960m, 2929m, 1658s, 1597s, 1556s, 1489s, 1438s, 1396s, 1315s, 1284m, 1238s, 1109s, 1006s, 987s, 941s, 839s, 808s, 765s, 684s, 669s, 580w. Anal. Calcd for $C_{168}H_{144}Co_7N_{12}O_{40}$: C, 59.63; H, 4.25; N, 4.96%. Found: C, 58.33; H, 5.09; N, 5.39%.

Synthesis of 2,4,6-tris(4-pyridyl)-1,3,5-triazine (TPT).

2,4,6-Tris(4-pyridyl)-1,3,5-triazine was prepared according to literature procedure.[S4]

Synthesis of 2,4,6-tris[1-(3-carboxylphenoxy)ylmethyl]mesitylene (H_3L).

2,4,6-Tris[1-(3-carboxylphenoxy)ylmethyl]mesitylene was prepared according to literature method.[S5]

Gas adsorption experiments

The solvated MOF samples were soaked in absolute ethanol to exchange the occluded solvent for C_2H_5OH for 48 h. The treated samples were loaded in sample tubes and activated under high vacuum (less than 10^{-5} Torr) at 110 °C. Degassed sample (78 mg) was then used for gas sorption measurements (CH_4 at 273 K, CO_2 at 273 K and 195 K), which were performed using an ASAP 2020 M gas adsorption analyzer. High pressure CO_2 gas adsorption measurement at room temperature was performed using a PCTpro-2000 high pressure adsorption analyzer and UHP-grade gases were used in measurements. The samples (138 mg) used in the measurement were first degassed on the degas station of ASAP 2020 M gas adsorption analyzer then transferred to the sample holder of high pressure adsorption analyzer and further degassed at 110 °C for five hours before the measurements. The CO_2 sorption isotherms were collected at 273 K in an ice water mixture bath and at 195 K in dry ice and acetone bath. The CH_4 sorption isotherms were collected at 273 K in an ice water mixture bath.

Gas adsorption properties

The measured adsorption isotherms showed that **1** adsorbed a small amount of CO_2 at 273 K and 1 atm (Figure S17a), while a saturated CO_2 uptake of about 20.55% can be obtained till 31 bar in the high pressure adsorption experiments (Figure S17b). The PXRD pattern of the activated **1** (at 110 ^{o}C) after gas adsorption agrees well with that of the as-synthesized sample, except for the slight low-angle PXRD peak shifts (2 Theta) due to the loss of solvents during the pre-activation process (Figure S18), indicating that the structure of **1** mainly retained in the course of gas adsorption. Notably, the CO_2 adsorption isotherm of the activated **1** subsequently measured at 195 K exhibits a type I isotherm in the low-pressure range (Figure

S17a), which can be seen an indication of the microporous structure of **1**. The Langmuir and BET surface areas of the activated sample measured from this isotherm were 104.3 and 75.8 m^2 g^{-1}, respectively.

Electrochemical Measurements

Electrochemical performances were evaluated in CR2025-type coin cells assembled in a high-purity argon-filled glove box with lithium as counter and reference electrodes. The working electrode was fabricated by mixing MOF, Ketjen black (KB) and poly(vinylidene fluoride) (PVDF) with the weight ratio of 70:20:10 to form slurry, and then coated onto copper foil. The mass of each electrode was approximately 1 mg. The electrolyte consisted of a solution of 1 mol L^{-1} $LiPF_6$ in dimethyl carbonate (DMC), ethylmethyl carbonate (EMC), and ethylene carbonate (EC) (1:1:1 v/v/v). Celgard 2400 membrane was used as a separator. Discharge/charge tests were performed galvanostatically between the potential range of 0.01-3.00 V (vs. Li/Li^+) under LAND-CT2001A battery testers. Cyclic voltammograms (CVs) were conducted on a Zahner-Elektrik IM6e electrochemical workstation recorded at a scanning rate of 0.1 mV s^{-1} between 0.01 and 3.00 V (vs. Li/Li^+) at room temperature.

[S1] V. A. Blatov, *IUCr CompComm Newsletter*, http://www.topos.samsu.ru. **2006**, *7*, 4.

[S2] G. M. Sheldrick, *SHELXTL NT*, Version 5.1 (Program for Solution and Refinement of Crystal Structures). University of Göttingen: Göttingen, Germany, **1997**.

[S3] A. L. Spek, *J. Appl. Crystallogr*. **2003**, *36*, 7.

[S4] H. L. Anderson, S. Anderson and J. K. M. Sanders, *J. Chem. Soc., Perkin Trans. 1* **1995**, *18*, 2231.

[S5] J. F. Eubank, H. Mouttaki, A. J. Cairns, Y. Belmabkhout, L. Wojtas, R. Luebke, M. Alkordi and M. Eddaoudi, *J. Am. Chem. Soc*. **2011**, *133*, 14204.

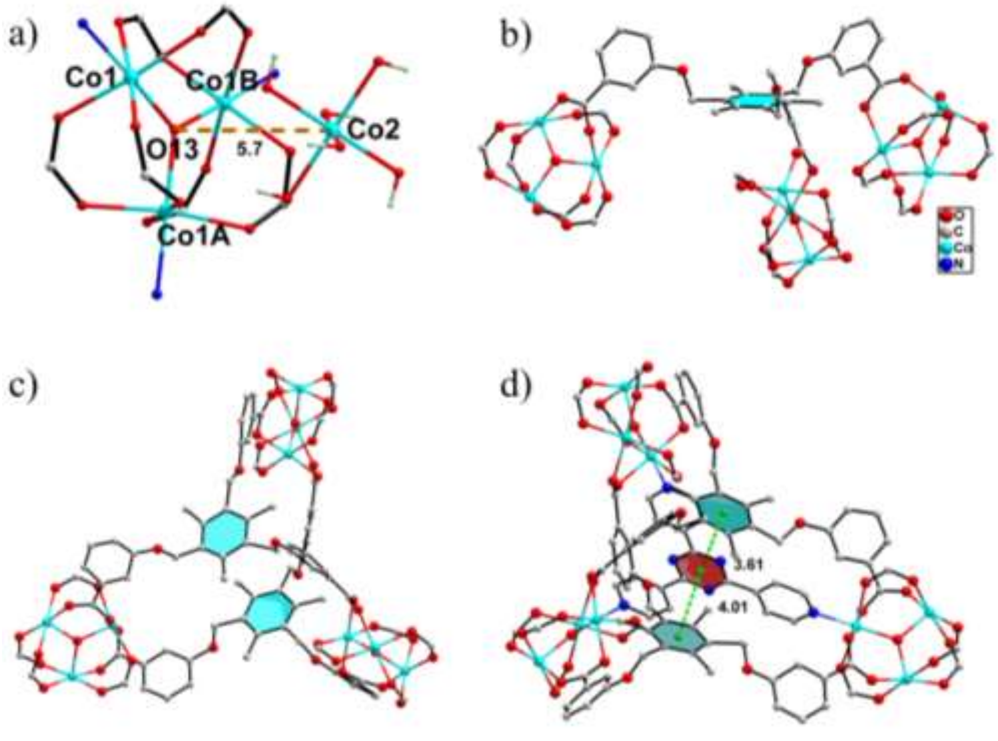

Figure S1. (a) Each $Co2^{2+}$ ion lies right above trinuclear Co_3 SBU. Symmetry codes: A: -x, -y, 1-z; B: -x, 1-y, 2-z; (b) The peripheral phenyl moieties orient perpendicular to the central benzene ring; (c) The formed hetero-triple-layered composite building block resembles a compressed chamber; (d) The weak π···π interaction (3.61 – 4.01 Å) between the central triazine ring of TPT and the central benzene ring of L^{3-}.

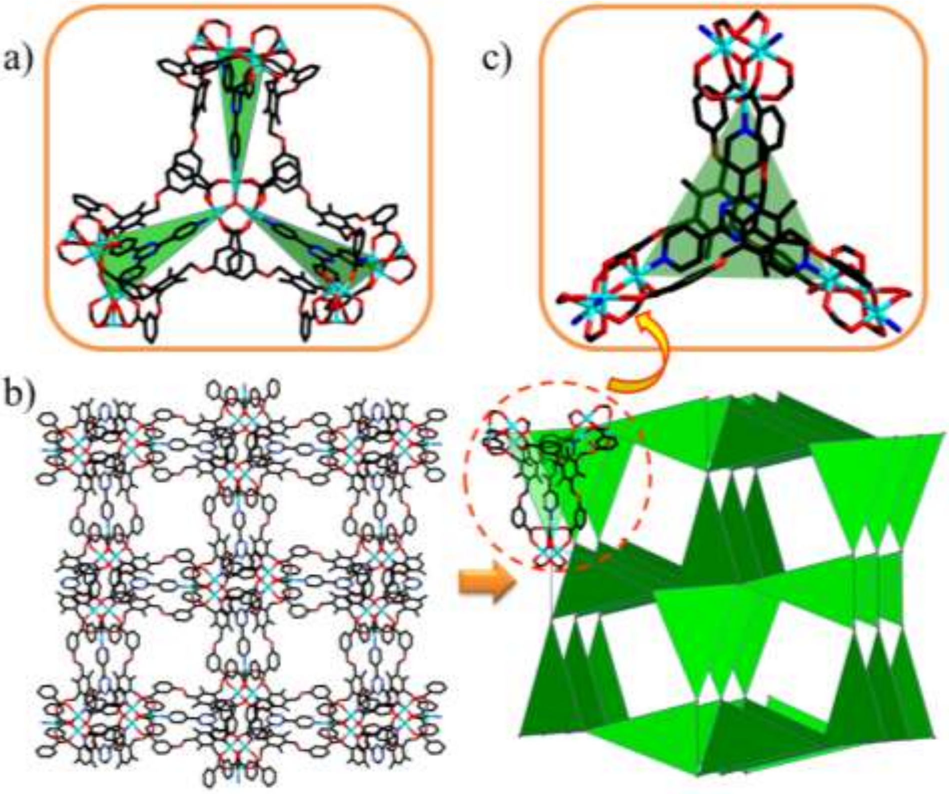

Figure S2. (a) A view of a propeller arrangement at Co_3 SBU; (b) The chiral 3D open framework; (**c**) A piece of the chiral propeller-type unit.

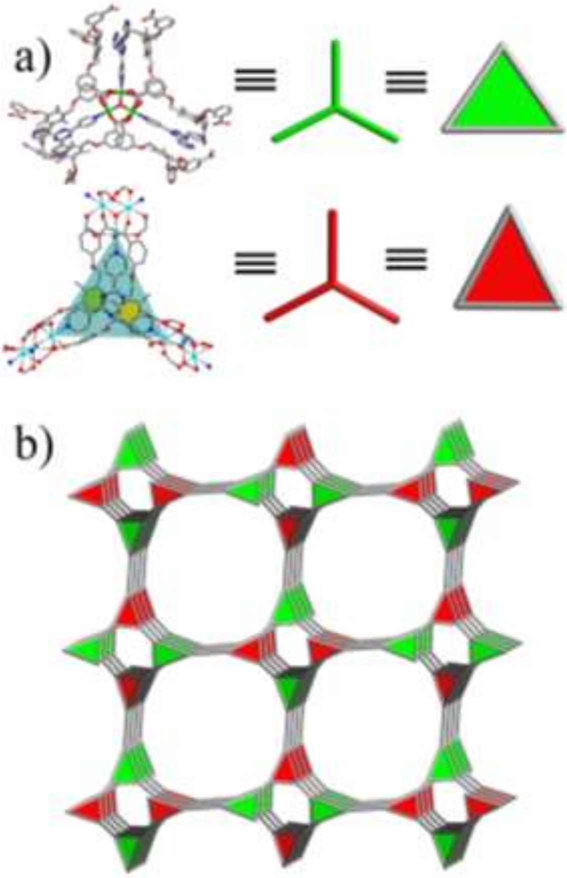

Figure S3. (a) The 3-connected nodes from trinuclear Co_3 SBUs and hetero-triple-layered building block L^{3-}-TPT-L^{3-}; (b) The chiral (10,3)-a net.

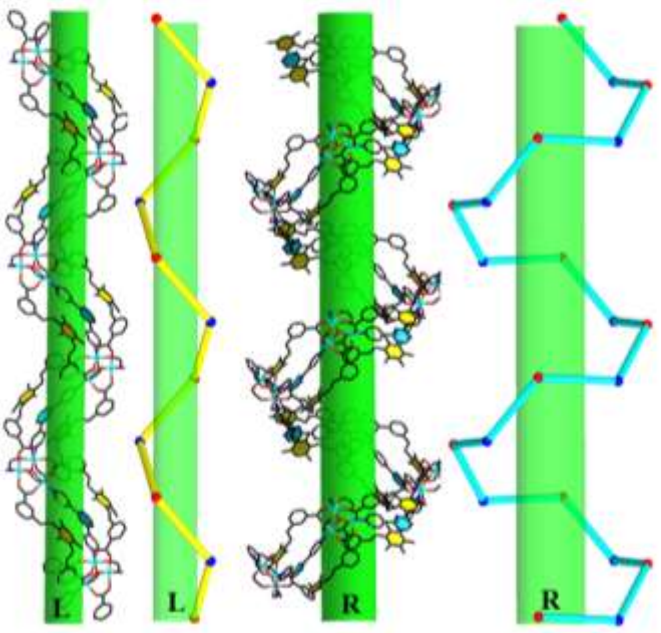

Figure S4. The left-handed and right-handed helical chains generated by SBUs threading hetero-triple-layered building blocks.

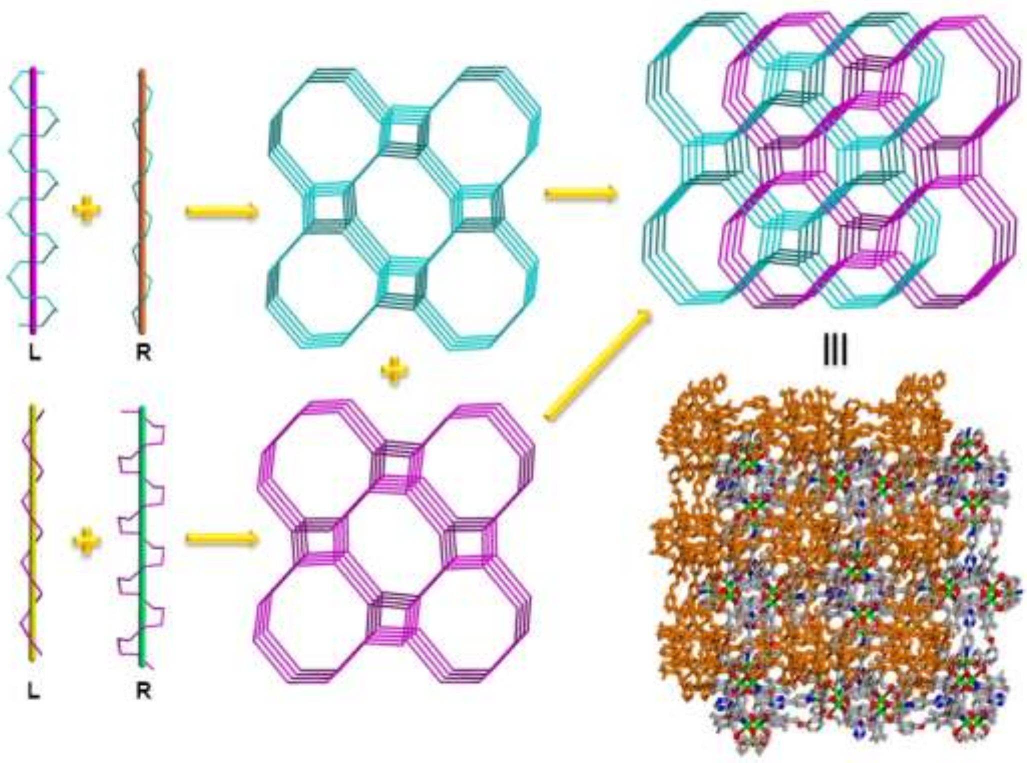

Figure S5. Schematic illustration of two-fold interpenetrated topology of the 3D network of **1**.

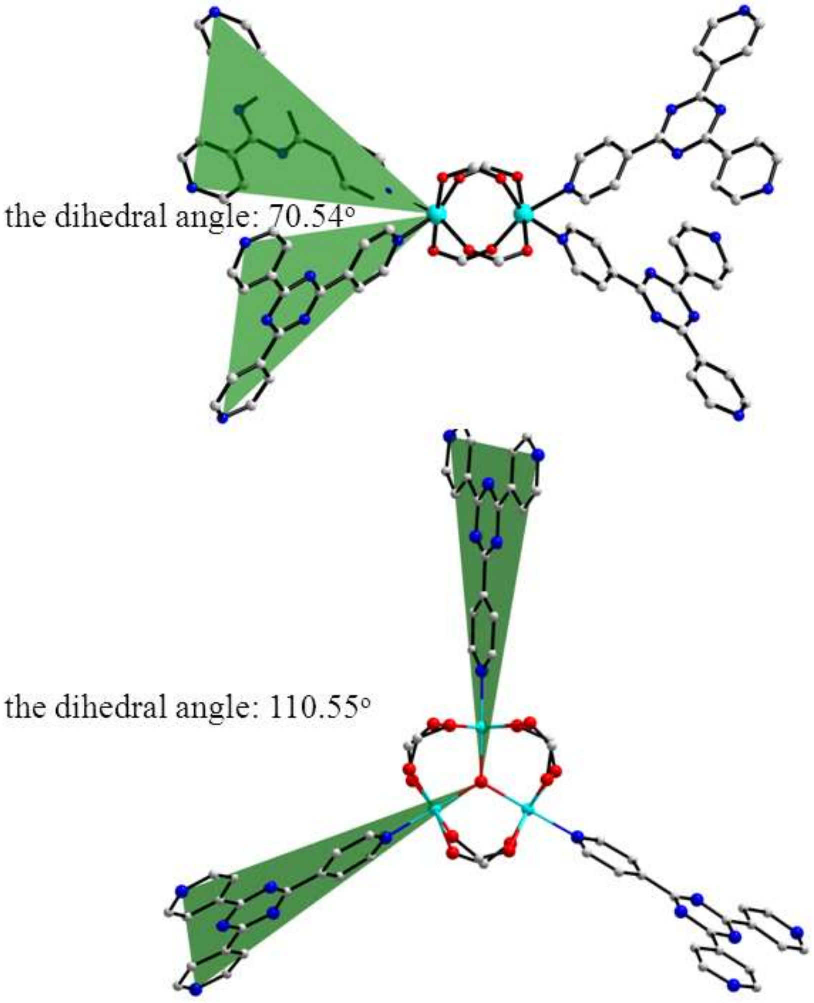

Figure S6. The dihedral angles between TPT ligands: 70.54° (up) for the double-walled MOP; 110.55° (down) for the triple-walled MOF. The coordinated L^{3-} units were omitted for clarity.

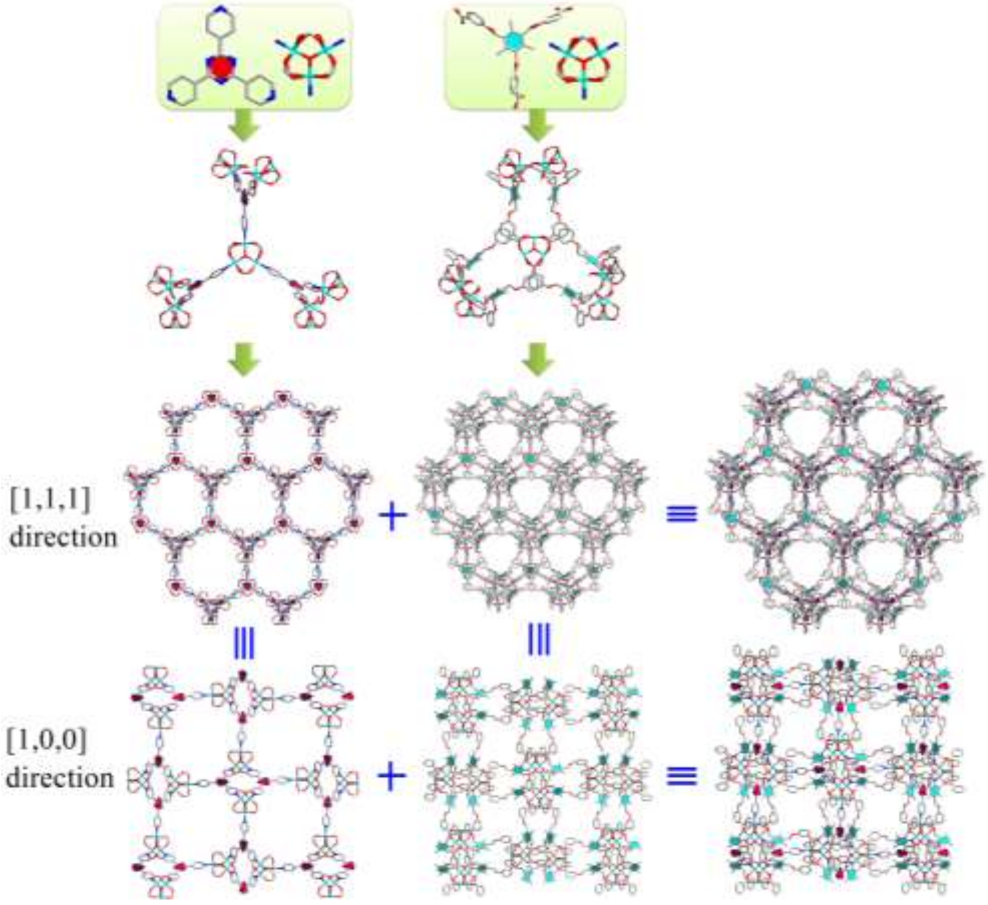

Figure S7. Structural analysis for the 3D framework of **1**.

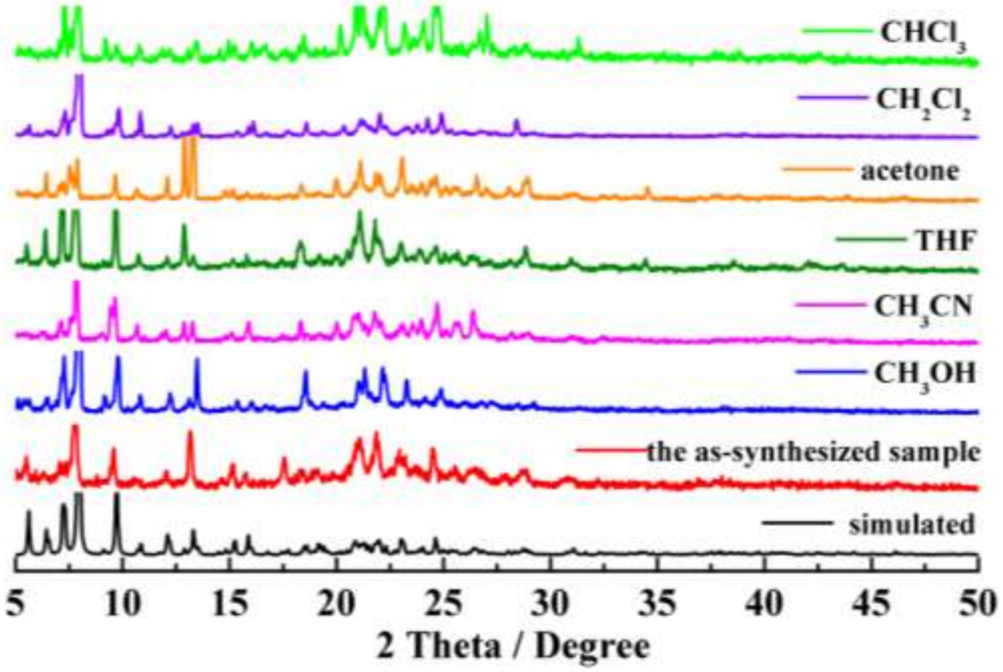

Figure S8. PXRD patterns of **1**: the as-synthesized pattern (red), the simulated pattern based on X-ray single-crystal data (black), and the patterns after **1** immersing in different solvents for 7 days.

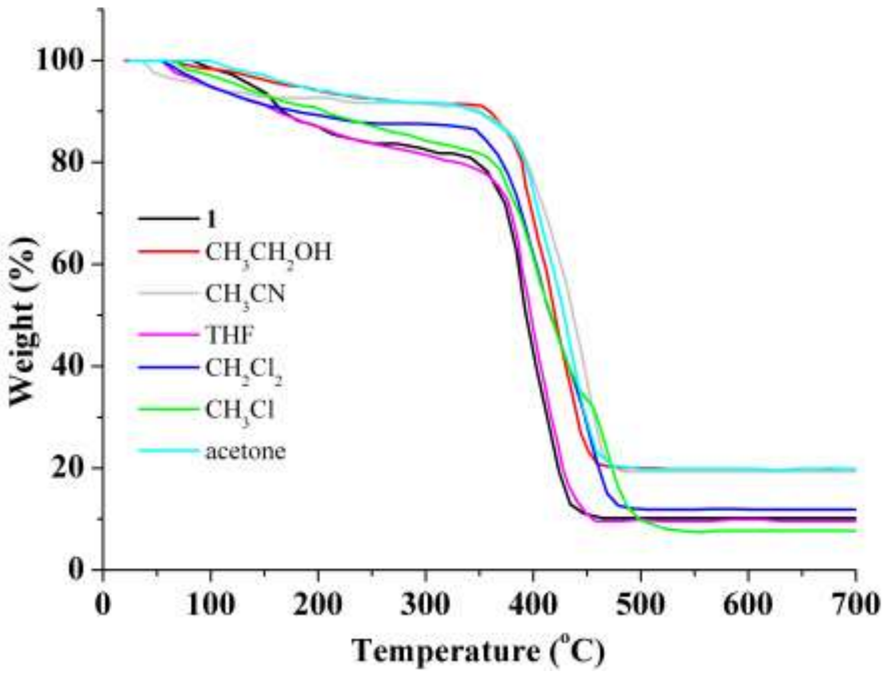

Figure S9. TGA curves of **1** and the crystalline samples immersed in different solvents for 7 days.

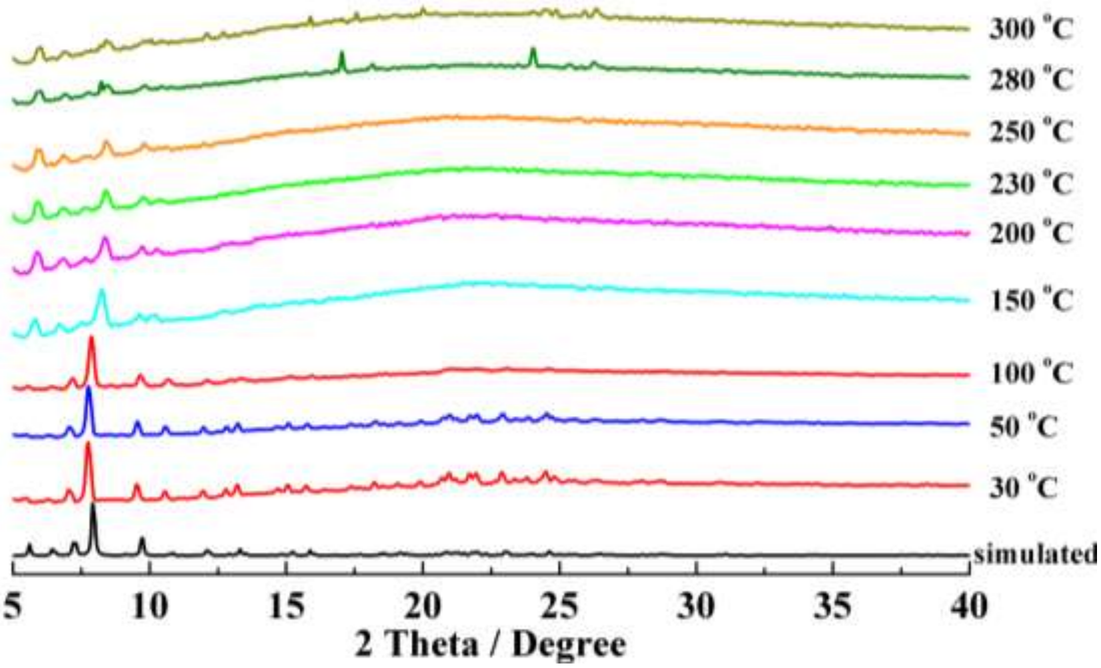

Figure S10. Variable-temperature PXRD patterns of the bulk sample of **1**.

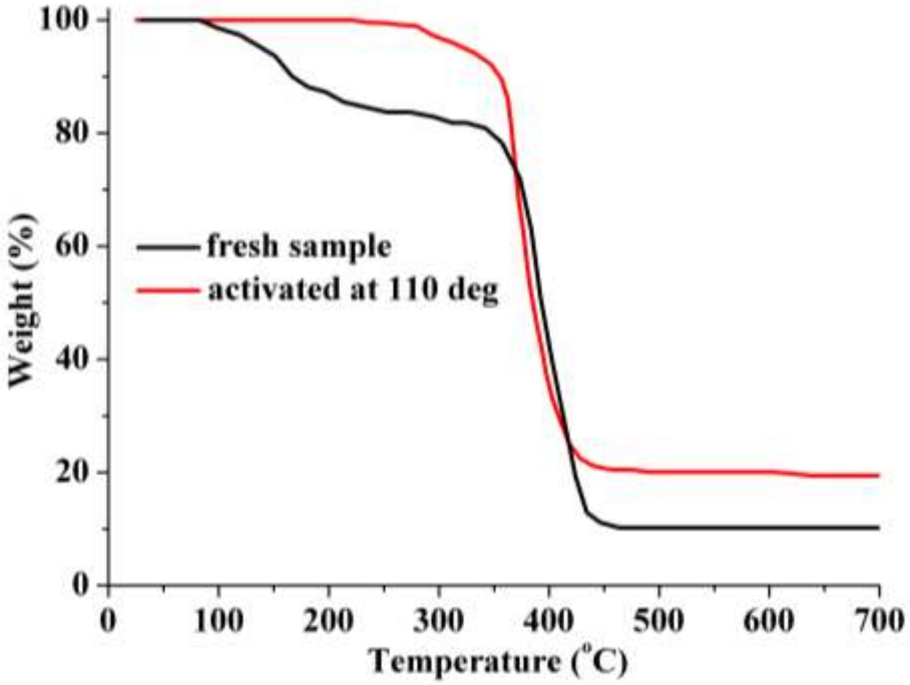

Figure S11. TGA curves of the as-synthesized and activated **1**.

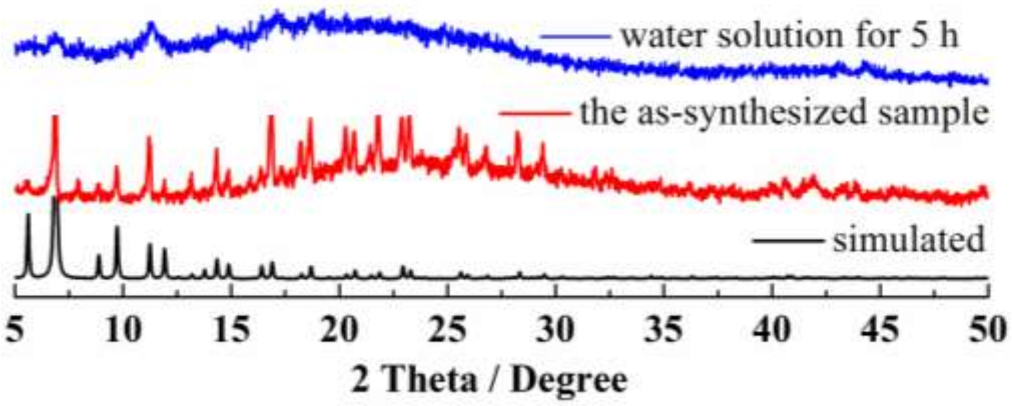

Figure S12. PXRD profiles for simulated, as-synthesized the double-walled ($[Co_3L_2(TPT)_2 \cdot xG]_n$ (*Angew*. *Chem*. *Int*. *Ed*. 2014, 53, 837-841), and $[Co_3L_2(TPT)_2 \cdot xG]_n$ soaked in water.

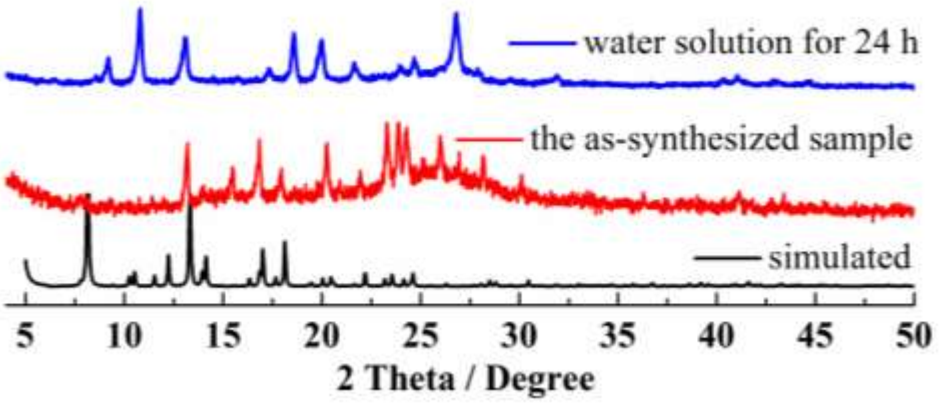

Figure S13. PXRD profiles for simulated, as-synthesized the single-walled $[(Co(SCN)_2)_3(TPT)_4 \cdot xG]_n$ (*J*. *Am*. *Chem*. *Soc*. 2014, *136*, 17899−17901), and $[(Co(SCN)_2)_3(TPT)_4 \cdot xG]_n$ soaked in water.

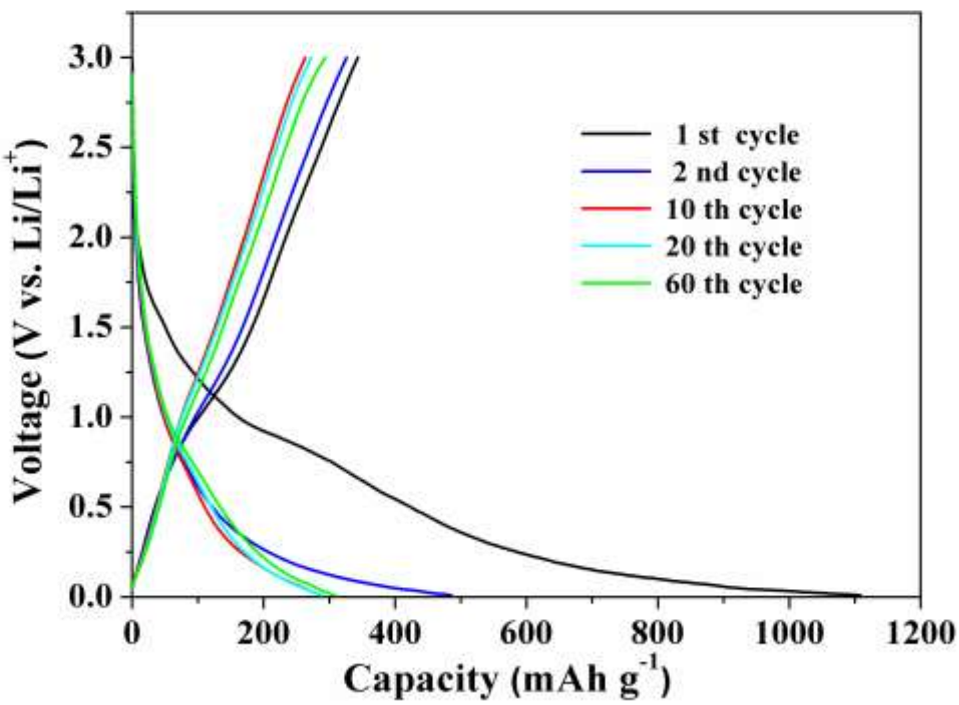

Figure S14. Discharge-charge plots of different cycles at a current density of 50 mA g^{-1}.

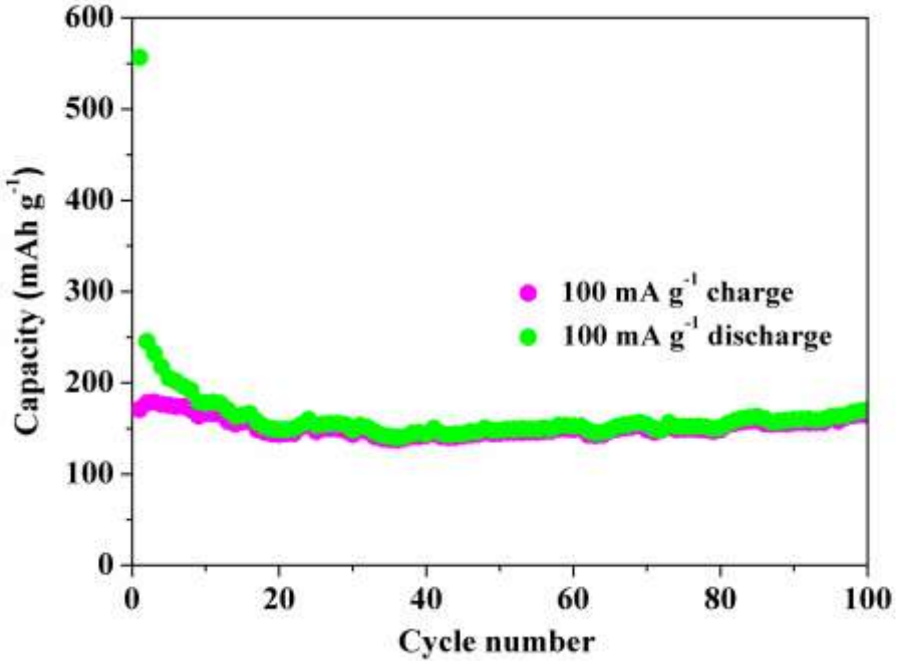

Figure S15. Cycling performance of **1** at a current density of 100 mA g^{-1}.

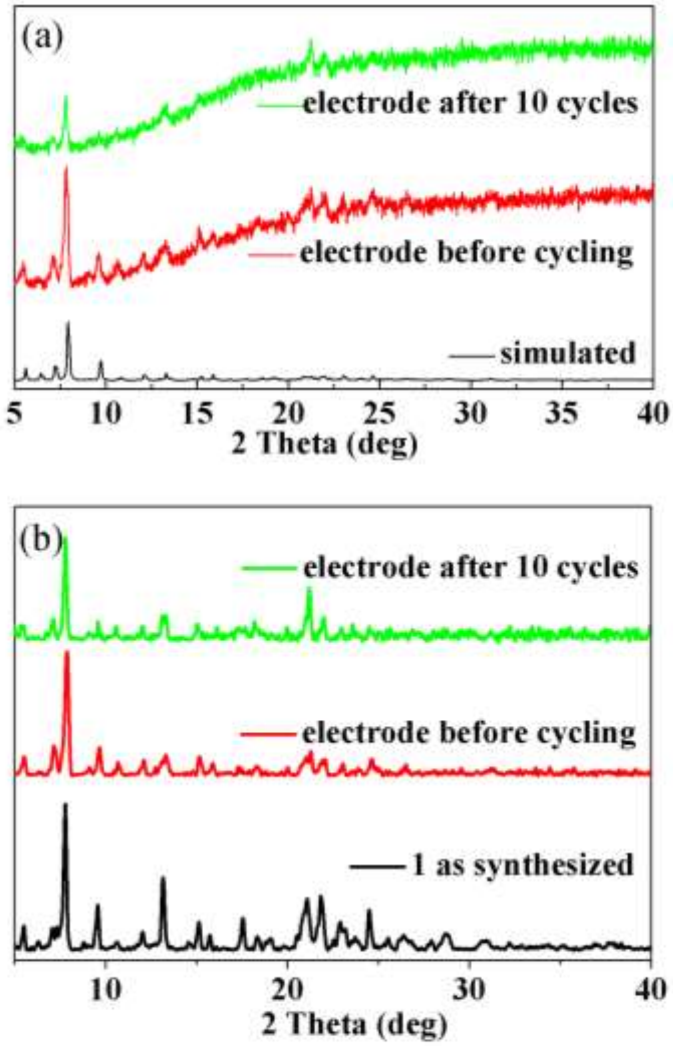

Figure S16. (a) The original XRD patterns of **1** as anode material during the charge-discharge cycling, with the simulated result from the single-crystal data as reference; (b) The XRD data treated by the Search-Match program to subtract the background.

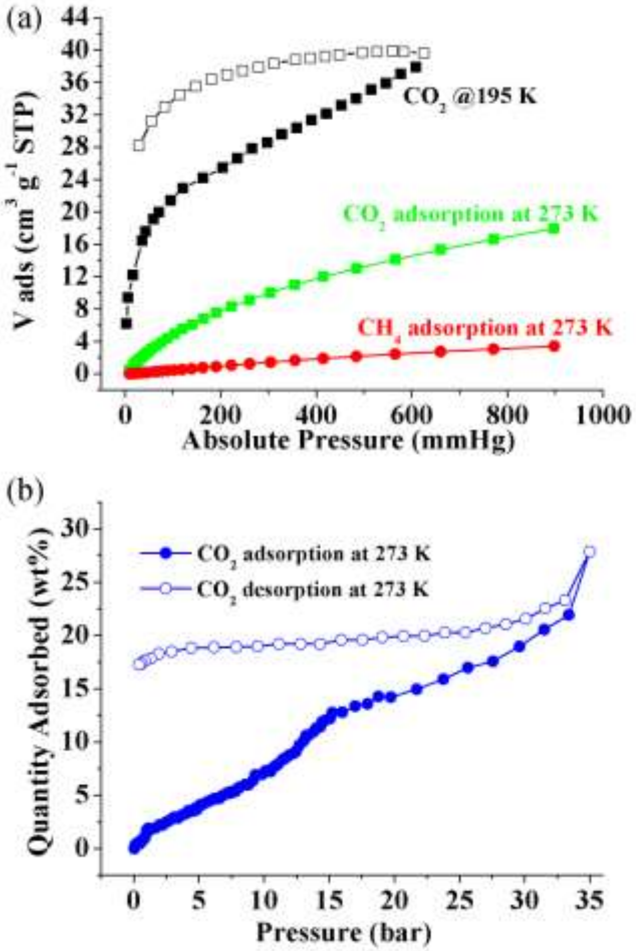

Figure S17. Gas adsorption-desorption isotherms: (a) CO_2, CH_4 at 273 K and CO_2 at 195 K for complex **1**. (b) High pressure CO_2 adsorption-desorption isotherms at 273 K.

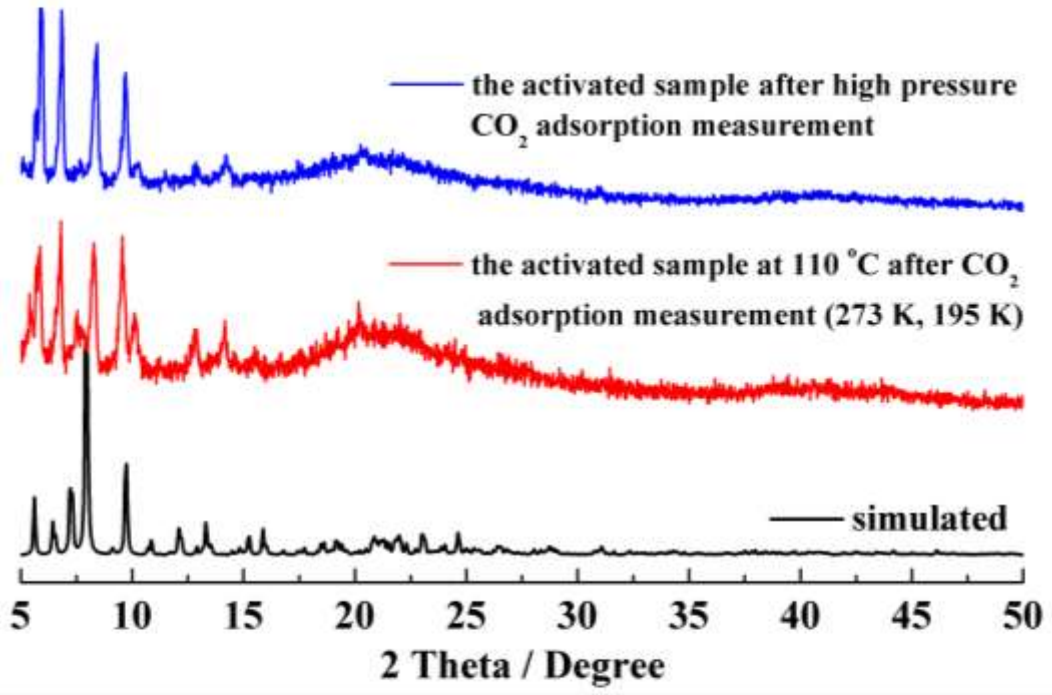

Figure S18. PXRD patterns of **1**: the simulated pattern based on X-ray single-crystal data (black), the activated sample at 110 °C after CO_2 adsorption measurements (red) and after high pressure CO_2 adsorption measurement (blue).
